# Miliary Tuberculosis: A Case Report Highlighting the Diagnostic Challenges Associated With the Condition

**DOI:** 10.7759/cureus.29339

**Published:** 2022-09-19

**Authors:** Usman Ilyas, Abrahim Mahmood, Amee M Pansuriya, Zaryab Umar, Ian Landry

**Affiliations:** 1 Internal Medicine, Icahn School of Medicine at Mount Sinai, Queens Hospital Center, Jamaica, USA; 2 Internal Medicine, New York Institute of Technology College of Osteopathic Medicine, Flushing, USA; 3 Internal Medicine, New York Institute of Technology College of Osteopathic Medicine, Old Westbury, USA

**Keywords:** mycobacterium avium intracellulare, mycobacterium avium-complex, mycobacterium tuberculosis complex, granulomatous disease, “granuloma”, “pancytopenia”, end stage renal disease (esrd), miliary tuberulosis, disseminated tuberculosis

## Abstract

End-stage renal disease requiring chronic dialysis is an immunocompromised state which increases the risk of tuberculosis development and its spread. Due to the high frequency of non-specific or “decoy” symptoms at presentation and frequent extrapulmonary involvement, diagnosis of tuberculosis is a significant challenge. Therefore, it is correctly labeled as ‘Tuberculosis; the great imitator’ as it can mimic various other disease processes, causing confusion and testing of subsystems involved in the disease process, which come back as abnormal, leading to a vicious cycle. Missing the diagnosis leads to grave consequences, especially in a patient with a miliary form of tuberculosis, as the prognosis with any delay in treatment is poor. High diagnostic suspicion is required to promptly diagnose and treat the condition, especially in a resource-rich setting where tuberculosis is uncommon. Here, we report a patient with miliary tuberculosis who presented with a chief complaint of chronic diarrhea and fecal continence, with prior recent negative interferon-gamma release assay testing. Due to every organ system involved, multiple subspecialties were on board, with a broad differential in mind, including malabsorption syndromes, neoplasia, infections, amyloidosis, and autoimmune disorders, and therefore, numerous tests were performed. However, despite all efforts, the diagnosis was delayed significantly, leading to the unfortunate demise of the patient. The case report sheds light on unique clinical features of miliary tuberculosis, diagnostic findings, and a reminder to always keep tuberculosis high in the differential in an appropriate clinical setting.

## Introduction

Miliary tuberculosis (TB) continues to pose a great diagnostic challenge due to multiple modes of presentation, depending on the extent and severity of both pulmonary and extrapulmonary organ involvement [1]. Although miliary TB is rare, the risk of disease rises with immunocompromised states such as acquired immune deficiency syndrome, diabetes, end-stage renal disease (ESRD) on dialysis, and usage of corticosteroid or other immunosuppressive agents [2]. Gastrointestinal symptoms such as abdominal pain, nausea, vomiting, and diarrhea are uncommon clinical findings in miliary TB [3-5]. Here, we present a unique case of miliary TB in an ESRD patient with primarily gastrointestinal complaints at admission. Additional clinical and laboratory findings noted through the course of hospital stay included pancytopenia (including profound symptomatic thrombocytopenia), clotting factor abnormalities, hypoxemia, ascites, transaminitis, and recurrent fevers, which in the setting of a negative quantiferon tuberculosis test and positive preliminary cultures for Mycobacterium avium complex led to an incorrect diagnosis and treatment, leading to a death that could have been prevented.

## Case presentation

**Figure 1:**
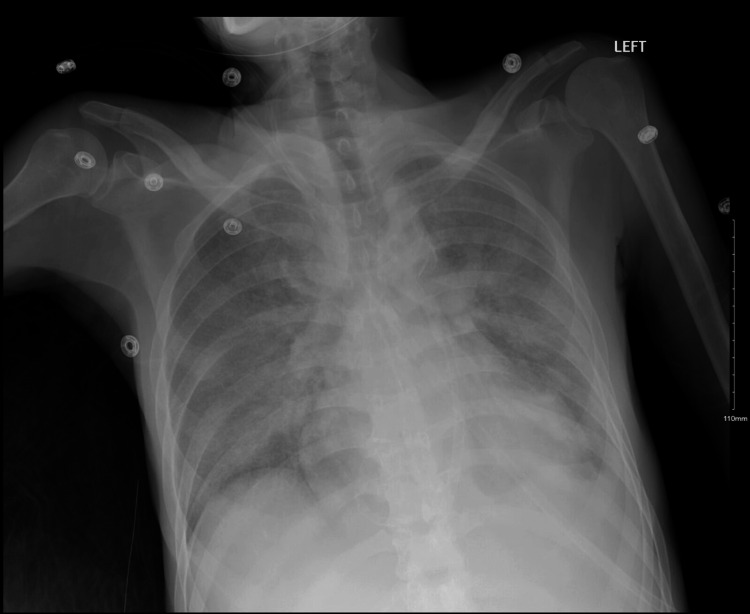
Chest x-ray showing diffuse ground-glass consolidation concerning for pneumonia or edema.

The patient was a 49-year-old man with a past medical history of type 2 diabetes, hypertension, ESRD on thrice weekly dialysis, coronary artery disease (drug-eluting stent in right coronary artery for 80% stenosis), heart failure with a reduced ejection fraction of 30-35%, and pulmonary hypertension with pulmonary artery systolic pressure of 65.76 mmHg who presented with complaints of six months of diarrheal episodes described as one time during the day and one time during the night, with stool being brown-green and of sticky and watery consistency. Over the last three months, he had a progressive loss of control over his bowel movements causing spontaneous episodes without him being able to anticipate them. Therefore, he had to resort to wearing adult diapers. He endorsed dry cough and subjective fevers as well. A day before presenting to the emergency department, he had an episode of falling when he became dizzy upon getting up from the sofa. He felt lightheaded, therefore, could not prevent the fall but denies losing consciousness. His physical examination in the emergency department showed bilateral coarse crackles, soft, mildly distended abdomen with positive bowel sounds and bilateral 2+ pedal edema. On admission, lab results showed pancytopenia (Tables 1, 2). A chest x-ray is shown in Figure 1.

**Table 1:** Patient's complete blood count throughout the hospital stay.

Lab with reference range and units	1 year before admission	6 months before admission	Day of admission	Day 7 of admission	Day 14 of admission	Day 21 of admission	Day 28 of admission	Day 35 of admission	Day 42 of admission
Hemoglobin (14.0-18.0 g/dL)	11	10.9	8.8	7.9	6.8	7.7	9.2	8.5	8.0
Platelet (150-450 x10^3/mcL)	162	144	72	30	36	54	13	9	22
White blood cell count (4.8-10.8 x10^3/mcL)	9.09	5.36	2.84	4.99	4.44	6.26	10.89	15.65	9.04
Absolute neutrophil count (2.1-7.60x10^3/mcL)	5.19	4.2	2.39	4.26	3.65	5.58	10.34	15.25	8.7
Absolute lymphocyte count (1.00-4.9x10^3/mcL)	1.37	0.41	0.17	0.24	0.27	0.16	0.24	0.12	0.04
Absolute monocyte count (0.1-1.1x10^3/mcL)	0.74	0.56	0.23	0.41	0.4	0.39	0.11	0.15	0.19
Absolute eosinophil count (0.1-0.4x10^3/mcL)	1.75	0.17	0.02	0.03	0.08	0.09	0.03	0.00	0.00
Absolute basophil count (0.0-0.2x10^3/mcL)	0.03	0.01	0.01	0.01	0.02	0.02	0.10	0.01	0.02

**Table 2:** Basic metabolic panel, liver function tests, and inflammatory markers on admission

Lab	Patient’s value	Reference range and units
Sodium	124	136-145 mmol/L
Potassium	4.1	3.5-5.1 mmol/L
Chloride	84	98-108 mmol/L
Bicarbonate	21	22-29 mmol/L
Blood urea nitrogen	56	6-23 mg/dL
Creatinine	6.44	0.50-1.20 mg/dL
Calcium	9.7	8.6-10.3 mg/dL
Albumin	2.4	3.5-5.2 g/dL
Total protein	6.6	6.6-8.7 g/dL
Total bilirubin	0.9	0.0-1.2 mg/dL
Direct bilirubin	0.3	0.0-0.3 mg/dL
Alanine aminotransferase	32	0-41 U/L
Aspartate aminotransferase	15	5-40 U/L
Alkaline phosphatase	183	40-129 U/L
Prothrombin time	12.8	10-13 seconds
International normalized ratio	1.1	1.4 ratio
Activated partial thromboplastin time	34	25.1-36.5 seconds
Procalcitonin	2.18	0.02-0.10 ng/mL
Hemoglobin A1C	6.3	4-5.6%
Erythrocyte sedimentation rate	41	0-10 mm/hr
C-Reactive protein	98.3	0-5 mg/L

He was admitted with the suspicion of diarrhea secondary to infectious versus malabsorptive etiology with malabsorption higher on the differential, given the chronic nature of his symptoms and lack of fever or leukocytosis. However, a complete workup, including stool routine examination, stool bacterial culture, blood cultures, Giardia, Clostridium difficile, human immunodeficiency virus, Strongyloides, stool leukocytes, stool calprotectin, stool ova and parasites, celiac workup, stool fat, and stool elastase, was sent and came back negative (Table 3).

**Table 3:** Stool Studies for infectious and inflammatory causes.

Lab	Patient’s value	Reference range and units
Fecal leukocyte count with wright stain	Negative	Negative
Ova and parasite screen stool	Negative	Negative
Giardia antigen	Negative	Negative
Clostridium difficile antigen	Negative	Negative
Clostridium difficile toxin A/B	Negative	Negative
Immunoglobulin A	297	84-499 mg/dL
Transglutaminase antibody Immunoglobulin A	<1.2	0-3.9 U/mL
Fecal occult blood test	Negative	Negative
Stool fats neutral, and total	<60 and <100 droplets /high power field	<60 and <100 droplets / high power field
Calprotectin, fecal	103	0-120 ug/g
Pancreatic elastase, stool	412	>200

**Figure 2:**
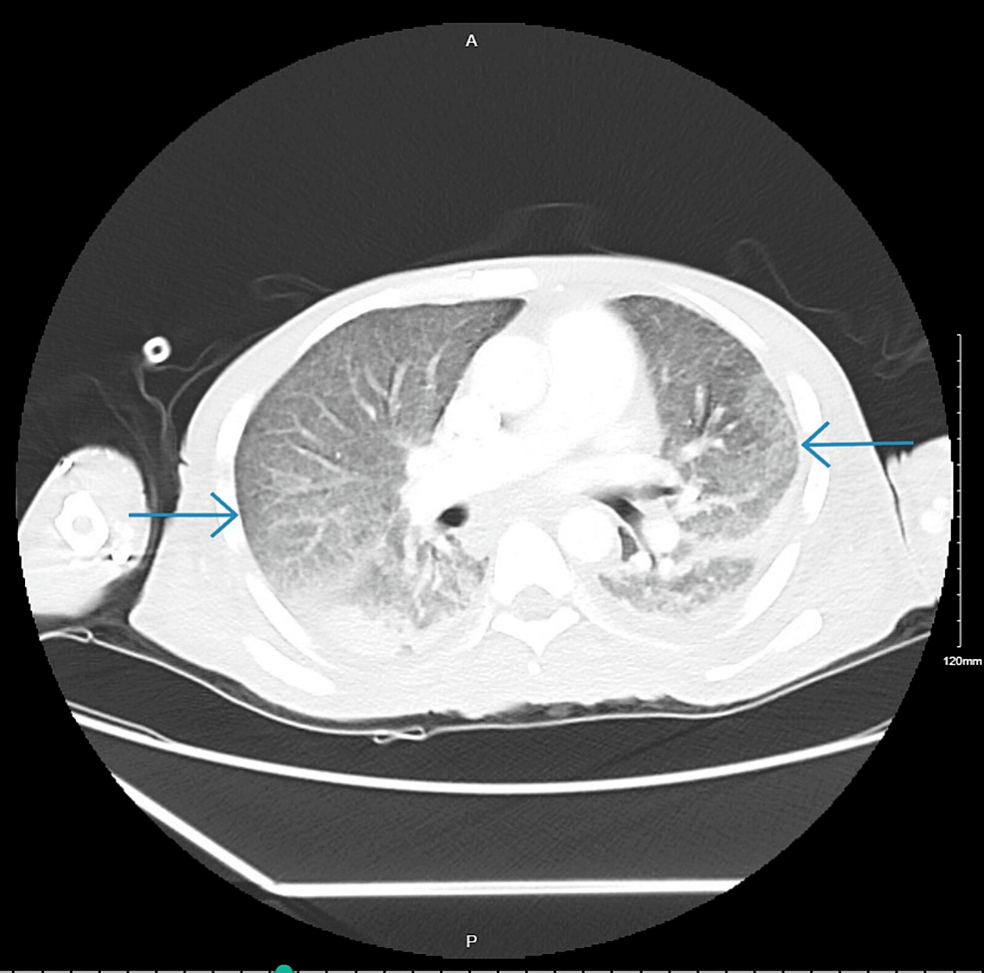
CT chest with contrast showing diffuse bilateral ground-glass opacities (blue arrows).

**Figure 3:**
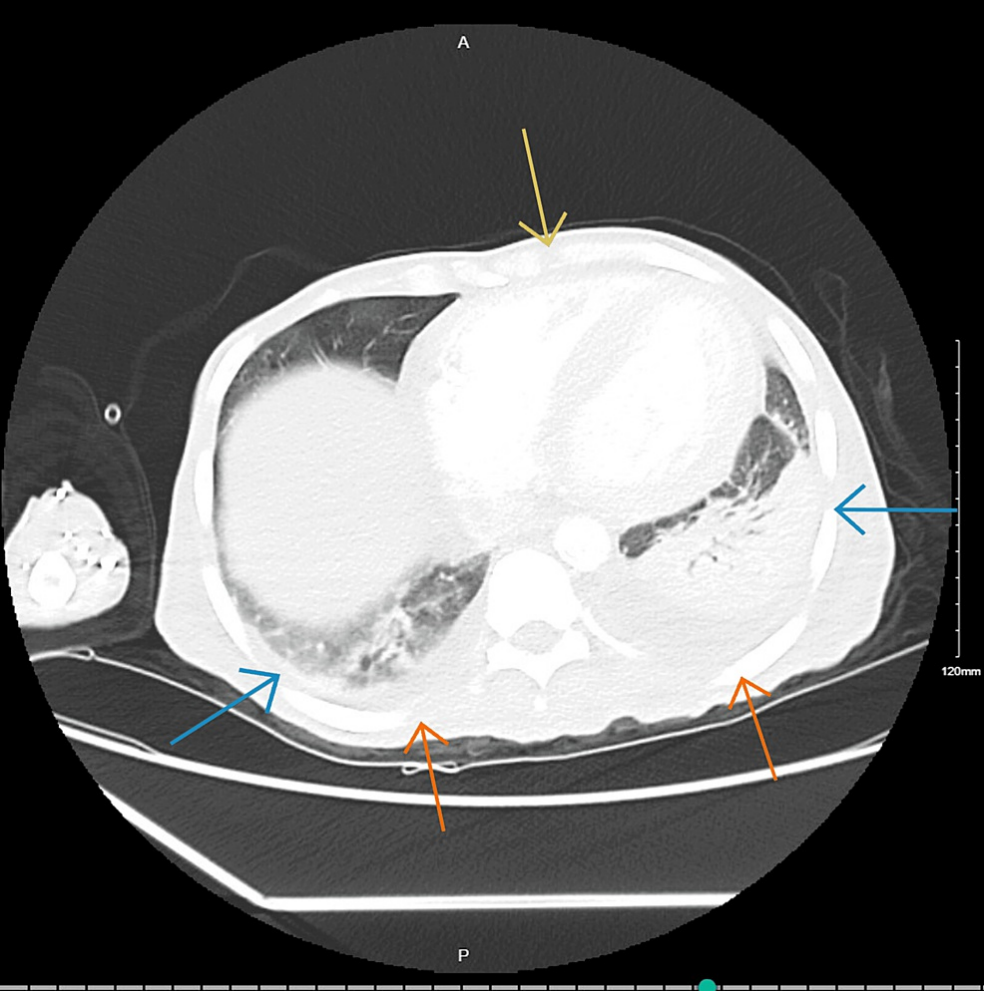
CT chest with contrast showing consolidations within lower lungs (blue arrows), cardiomegaly (yellow arrow), and bilateral pleural effusions(orange arrows).

A computed tomography (CT) scan of the abdomen and pelvis showed mild abdominopelvic ascites. On the fourth day of hospitalization, he had a sharp drop in hemoglobin from 8.8 to 7.1 with mild nasal bleeding. Heparin prophylaxis was stopped, intermittent pneumatic compression devices were placed, and a fecal occult blood test was sent. The patient also started desaturating to 88% oxygen on room air, for which 3 liters of oxygen per nasal cannula was started, maintaining oxygen saturation above 94%. Chest CT with contrast is shown below (Figures 2, 3).

**Figure 4:**
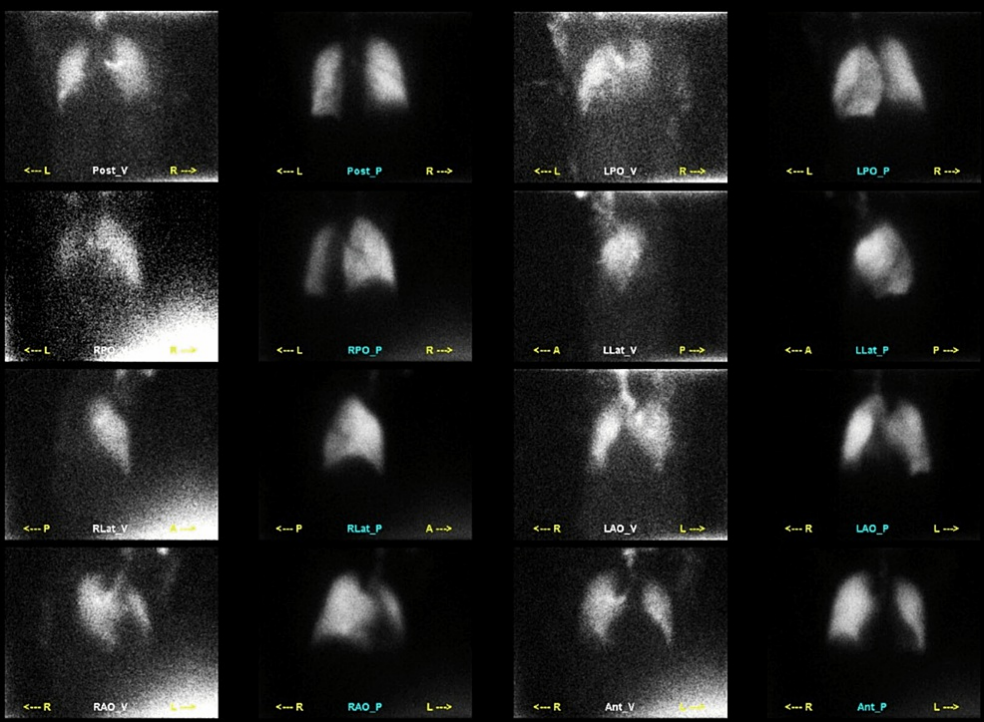
Nuclear medicine lung ventilation-perfusion scan shows matching ventilation/perfusion images, consistent with low probability for pulmonary thromboembolism.

Hypoxic respiratory failure secondary to fluid overload was assumed based on CT findings. An echocardiogram showed a left ventricle ejection fraction of 30-35% with grade 3 left ventricular diastolic dysfunction, pulmonary artery systolic pressure of 65.76 mmHg, and severely elevated central venous pressure of 11-20 mmHg; therefore, more aggressive dialysis with fluid removal was planned. A nuclear medicine ventilation-perfusion scan ruled out pulmonary embolism (Figure 4).

A gastroenterology consult recommended a colonoscopy, considering his diarrheal episodes and lower gastrointestinal bleeding. For thrombocytopenia with platelet count drop from 72,000 per mcL to 35,000 per mcL in five days, heparin-induced thrombocytopenia workup with platelet factor 4 was sent due to a 4T score for heparin-induced thrombocytopenia of 4 with intermediate probability and came back negative. Heparin use, including for dialysis, was avoided. Piperacillin tazobactam was stopped as it was considered the likely cause of recurrent hypokalemia and thrombocytopenia. Peripheral smear showed polychromasia and burr cells but no schistocytes. An antinuclear antibody screen for autoimmune conditions was negative. Complete anemia workup results are shown in Table 4. 

**Table 4:** Anemia work-up, anti-nuclear antibody screen, and heparin-induced thrombocytopenia labs.

Lab	Patient’s value	Reference range and units
Vitamin B12	1568	232-1245 pg/mL
Serum Folate	2.5	>4.7 ng/mL
Lactate dehydrogenase	340	135-225 U/L
Haptoglobin	75	34-200 mg/dL
Reticulocyte count	2.41	0.5-1.5%
Reticulocyte absolute	0.0757	0.0221-0.0963x10^6/mcL
Red blood cell count	3.14	4.7-6.1x10^6/mcL
Heparin-platelet factor 4 antibody	<0.6	0.0-0.9 u/mL
Platelet antibodies Human leukocyte antigen Class I Antibody, Platelet IIB/IIIA Antibody, Platelet IB/IX Antibody, Platelet IA/IIA Antibody, Glycoprotein IV Antibody	Negative	Negative
Antinuclear antibodies	Negative	<1:80
Complement C3, serum	68	81-157 mg/dL
Complement C4, serum	29	13-39 mg/dL
Serum Protein Electrophoresis		
Total Protein	5.8	6-8.3g/dL
Albumin	2.3	3.6-5.5 g/dL
Alpha 1 Globulin Fraction	0.5	0.1-0.4 g/dL
Alpha 2 Globulin Fraction	0.5	0.5-1.0 g/dL
Beta Globulin Fraction	0.6	0.5-1.0 g/dL
Gamma Globulin Fraction	1.9	0.6-1.6 g/dL
Monoclonal spike (M-spike)	Two gamma migrating paraproteins identified (one IgG Lambda and one IgG Kappa band)	
Immunoglobulin Free Light Chain		
Immunoglobulin Kappa	18.52	0.33-1.94 mg/dL
Immunoglobulin Lambda	30.95	0.57-2.63 mg/dL
Kappa Lambda Ratio	0.60	0.26-1.65 Ratio

On the sixth admission day, he had an episode of altered mental status in the setting of a high-grade fever of 102.9 °F off antibiotics. The fever came down with acetaminophen. Repeat blood cultures and stool for acid-fast bacilli were sent. CT of the head was normal. Serum procalcitonin was elevated to 5.73 ng/mL (normal range 0.02-0.10 ng/mL). Vancomycin and cefepime were started, and he remained afebrile on antibiotics, with procalcitonin trending down to 2.73 ng/mL. He also had recurrent self-limiting nasal bleeds. The goal was to give cryoprecipitate if fibrinogen was below 120 mg/dL (normal range 200-393 mg/dL) and platelets transfusion if platelet count was below 20,000/mcL without bleed and 30000/mcL with bleed. Colonoscopy with biopsy was unremarkable for both gross and microscopic pathology.

**Figure 5:**
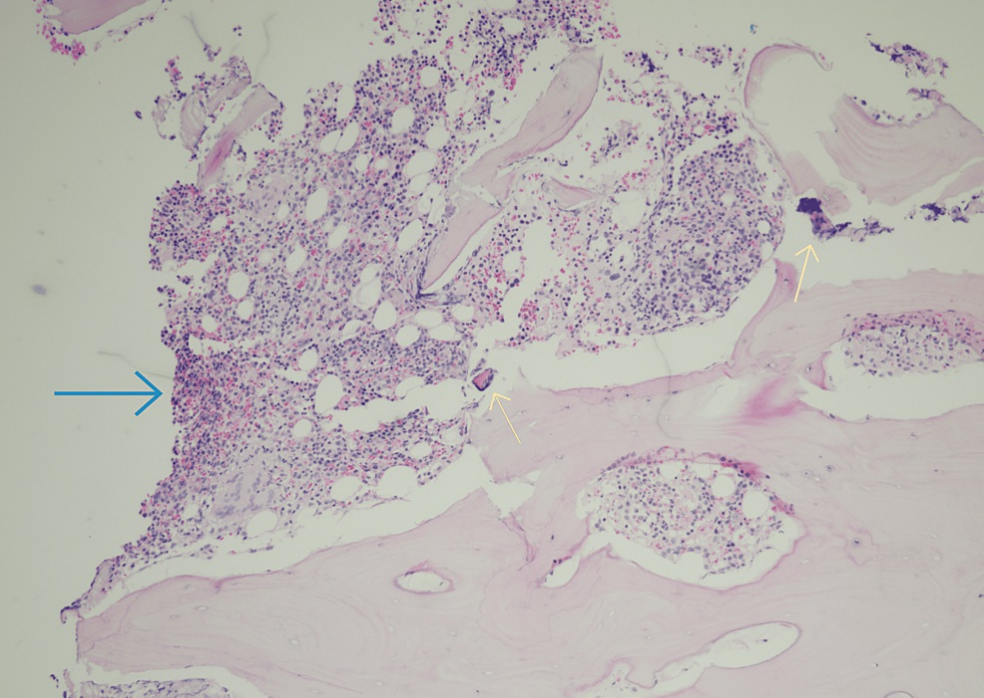
Bone marrow biopsy showing a small, subcortical sample containing normocellular marrow (60%), with trilineage hematopoiesis (blue arrow), full maturation, and mildly increased eosinophils. There are two small granulomas(yellow arrows) with associated multinucleated giant cells and foamy histiocytes (suggestive of lymphogranuloma). No evidence of myelodysplasia. The bony trabeculae are unremarkable. There is insufficient bone marrow for the evaluation of the fibrosis. Ziehl-Neelsen and Grocott's methenamine silver staining was negative.

A bone marrow biopsy (Figure 5) was done to find the cause of thrombocytopenia along with ruling out malignancy. Flow cytometry of the bone marrow aspirate was unremarkable; however, chromosome analysis showed an interstitial deletion of the long arm of chromosome 20 (20q deletion).

Due to frequent nosebleeds with low platelet count and low fibrinogen requiring platelets and cryoprecipitate, respectively, coagulation factors and mixing studies were obtained (Table 5), keeping in mind that although factor 8 deficiency is most common in amyloidosis, low levels of all factors have been reported previously.

**Table 5:** Coagulation factors and tumor markers obtained to rule out amyloidosis and malignancy, respectively.

Lab	Patient’s value	Reference range and units
Activated partial thromboplastin time (APTT)	45.6	25.1-36.5
Protime	15.1	10-13 seconds
International Normalized Ratio	1.3	
Fibrinogen	152	200-393 mg/dL
Dilute Russel Viper Venom time(DRVVT)		
Screen/Confirm ratio	0.80	0.00-1.21 Ratio
DRVVT Interpretation	LA Neg	LA neg
DRVVT Screen	49.5	<44 seconds
DRVVT Confirmatory	46.0	<44 seconds
Factor XII Assay	32	45-150%
Factor XI Assay	40	70-145%
Factor X Assay	56	70-170%
Factor IX Assay	57	80-165%
Factor VIII Assay	202	60-125%
Cancer antigen 125 (CA-125)	41	<38 U/mL
Carcinoembryonic antigen (CEA)	7.4	0.0-3.8 ng/mL
Cancer Antigen 19-9	449	<35 U/mL
Beta-2 Microglobulin	72.8	0.8-2.2 mg/mL

**Figure 6:**
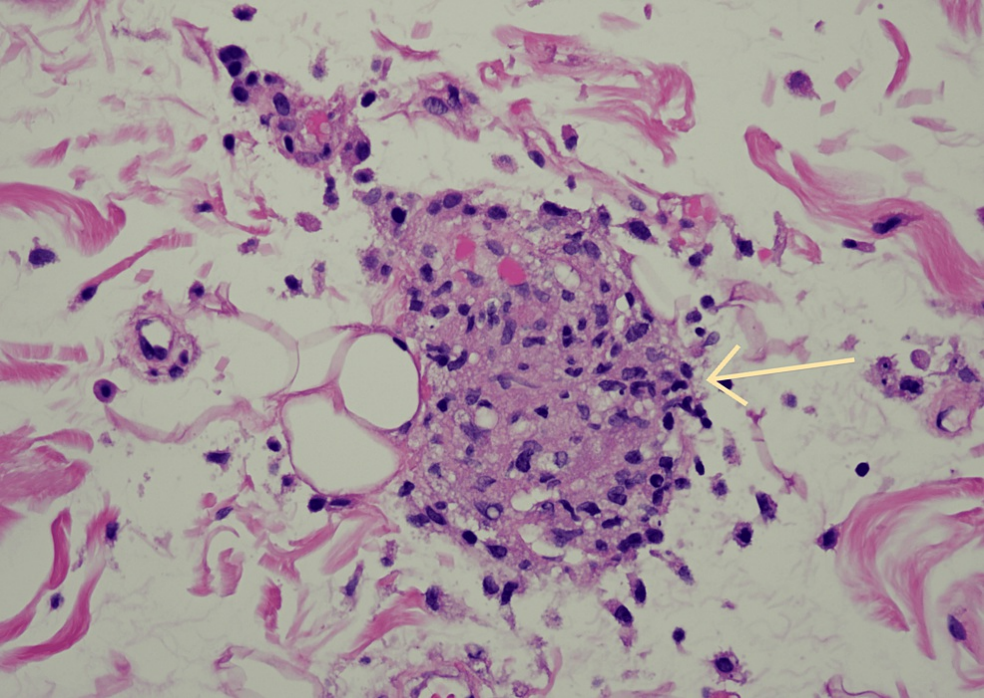
Abdominal fat pad biopsy showing fibrovascular and adipose tissue with a single multi-nucleated giant cell (yellow arrow). Amyloid or Congo red staining was negative.

A rectal fat pad biopsy (Figure 6) was performed to rule out amyloidosis, considering abnormal serum protein electrophoresis.

After completion of 10 days of cefepime and vancomycin, antibiotics were stopped. However, he spiked a fever of 102 ℉ again in the 48 hours, and antibiotics were resumed. A repeat serum procalcitonin came back as 9.86 ng/mL (normal range 0.02-0.10 ng/mL). His diarrhea restarted as well. Stool studies for Microspora, Isospora, Cryptosporidium, and Cyclospora, and blood cultures with blood tests for blood parasites, Babesia, Ehrlichia, and Anaplasma were sent and all came back negative. In the fourth week of admission, his acid-fast bacilli stool came back positive for Mycobacterium avium complex (MAC), however, it was deemed a contaminant at that time.

On the 30th day of admission, he was found to have an altered mental status. Glucose was undetectable with a blood pressure of systolic 80 by diastolic 40 mmHg and mean arterial pressure below the 60s. After 50% dextrose, glucose increased to 184, and blood pressure normalized with one albumin with 1 liter of normal saline. Dextrose water (5%) intravenous was started. The CT head was negative. Hypoglycemia secondary to poor oral intake and chronic condition was suspected with altered mental status due to hypoglycemia. He remained on the medical floor as he was not deemed a candidate for the intensive care unit (ICU). Hydrocortisone 100 mg every eight hours was started. Although his mental status improved temporarily, it later declined, and he became hypothermic. On the 31st day, he had a cardiac arrest. Advanced cardiac life support protocol was followed, with the return of spontaneous circulation achieved after 29 minutes of high-quality cardiopulmonary resuscitation with an initial rhythm of asystole. The patient was transferred to the ICU.

Suspecting a disseminated MAC infection, he was started on meropenem with foscarnet added after cytomegalovirus deoxyribonucleic acid (DNA) polymerase chain reaction of a blood sample returned as positive. He remained intubated for 15 days, with his code status changed to do not resuscitate after a family discussion regarding goals of care. Repeat blood and fungal cultures were sent. Electroencephalogram showed a predominant posterior rhythm consisting of diffuse and generalized bursts of the delta, theta, and sharply contoured activity followed by periods of relative cerebral inactivity, consistent with the hypoxic injury. Brainstem reflexes were absent with a Glasgow coma scale of 3/15. He had recurrent fever spikes of up to 104℉. On the 45th day, his vital signs began to decline despite maximal support until a flat line was seen on an electrocardiogram with fixed and dilated pupils. Approximately three weeks after his sad demise, acid-fast stool and blood tests returned positive for the rare Mycobacterium tuberculosis complex detected by a DNA probe and already detected Mycobacterium avium complex. A subsequent complete report documented a moderate mixed infection with pan-sensitive Mycobacterium tuberculosis and Mycobacterium avium complex.

## Discussion

John Jacobus Manget coined the word "miliary" in miliary tuberculosis in the year 1700. He used this word to describe the firm, small white nodules on the surface of an affected lung, similar to the appearance of millet seeds. Miliary TB is a rare manifestation of TB, and less than 2% of TB cases demonstrate miliary disease [6]. It is described as lymphohematogenous dissemination of M. tuberculosis, usually from central foci with subsequent infiltration into various organs, including the liver, bone marrow, spleen, lungs, and meninges, forming small caseating granulomas, about 1-3 mm in diameter [1]. Immunosuppression in the form of impaired cell-mediated immunity allows for the development and spread of TB [2,6]. Immunocompromised states leave the host more susceptible to the infection; examples of conditions include HIV/AIDS, diabetes, usage of corticosteroids or other immunosuppressive agents, or renal failure [2,6]. Patients like ours with ESRD on dialysis are an immunocompromised population and consequently are six to 25 times more likely to develop TB than the general population [7]. However, a history of TB is only present in 10-20% of the patients [1].

Miliary TB in a clinical setting typically presents insidiously with nonspecific symptoms related to the location of the bacterial infiltration. Common symptoms include weight loss, malaise, fever, and anorexia with or without respiratory symptoms (cough, dyspnea, pleuritic chest pain) [1]. Uremia is commonly associated with fatigue, malnutrition, and other nonspecific complaints, thereby concealing underlying TB disease [7]. Morning temperature spikes are a finding of diagnostic significance, mainly if it is of unknown origin, as they are only seen in three conditions: tuberculosis, typhoid fever, and periarteritis nodosa [1]. Several papers have reported the "damp shadow sign," or the sweat-drenched silhouette on the patient's bed, as a common clinical finding due to night sweats [8].

Gastrointestinal symptoms such as abdominal pain, nausea, vomiting, and diarrhea are uncommon clinical findings in miliary TB, as three retrospective series reported gastrointestinal complaints in approximately 21%, 12%, and 32% of patients [3-5]. However, our patient presented primarily with diarrheal symptoms for six months and gradually lost bowel movements over three months. Such a presentation prompted an extensive workup for an infectious vs. malabsorptive etiology.

The nonspecific symptoms of miliary TB require a high degree of suspicion to recognize the disease and order appropriate workup. Common lab abnormalities seen in TB are nonspecific. Erythrocyte sedimentation rate was elevated in approximately two-thirds of the cases in one study [2]. Arterial blood-gas analysis shows hypoxemia with a high alveolar-arterial gradient and hypocapnia [2]. Polyclonal gammopathy and hyponatremia are very common, with the latter likely from parenchymal lung disease with the inappropriate secretion of antidiuretic hormone [2]. Both were seen in our patient. Although thrombocytosis and leukocytosis are frequently present in pulmonary TB, miliary TB is associated with anemia of chronic disease, leukopenia, and thrombocytopenia [2]. Pancytopenia and coagulation abnormalities raised suspicion of malignancy as a source of fever in our patient with subsequent extensive workup.

Chest radiography classically demonstrates faint, reticulonodular, homogenous infiltrates with a relatively uniform distribution throughout the lungs [9-11]. In the early stages of the disease, however, radiograph findings may appear normal, making it challenging to diagnose miliary TB from chest radiographs alone [12]. Chest radiography has a sensitivity of 60-70% in miliary TB, which is influenced by the size of miliary calcifications, the astuteness of the radiologist, and the extent of underlying disease, which may make detection of the infiltrates more complex, leading to delayed or missed diagnosis with fatal consequences [1,2]. This form of the disease, termed cryptic miliary TB, is a diagnostic challenge even in areas where tuberculosis is endemic with high physician awareness [2]. In our resource-developed setting with low physician awareness, the absence of miliary calcifications and predominantly gastrointestinal symptoms on the presentation made diagnosis possible only retrospectively. High-resolution computed tomography (HRCT) of the chest is a more sensitive imaging test for miliary TB than plain chest radiography [13]. Disseminated nodules can be identified; however, these findings are sensitive and not specific to miliary TB. Imperative to add in the differential diagnosis include infectious (histoplasmosis, cryptococcosis, brucellosis, toxoplasmosis) and non-infectious diseases (sarcoidosis, lymphoma, metastatic disease), as they present with similar clinical findings.

A higher proportion of patients with miliary tuberculosis exhibit tuberculin anergy compared to those with pulmonary or extrapulmonary tuberculosis [6]. In addition to cross-reactivity with nontuberculous Mycobacteria and tuberculin positivity in Bacille Calmette-Guérin (BCG) vaccination, anergy makes tuberculin skin test a nondiagnostic test in miliary tuberculosis [6]. Similarly, in one study, interferon-gamma release assays such as quantiferon TB gold showed a negative result in 28.8% of patients with extrapulmonary tuberculosis, with sensitivity further being affected by HIV status, diabetes mellitus, neutropenia, immunosuppression, and severe TB disease [14]. This explains the two negative quantiferon TB gold tests in our patient three months before and at the presentation time.

Miliary TB can be diagnosed with either microbiological or histopathological tests. A microbiological examination of sputum is performed, which, if present, demonstrates a positive acid-fast stain due to the presence of mycolic acid in the cell wall of Mycobacterium [6]. The current clinical practice recommends three sputum specimens for acid-fast bacilli smear microscopy in all patients suspected of having pulmonary TB [15]. Sputum induction is advised in those unable to expectorate sputum [15]. In addition, performing a diagnostic nucleic acid amplification test on the initial respiratory specimen is recommended [15]. Mycobacterium blood cultures could be obtained if hematogenous dissemination is suspected. However, it is essential to identify the species as positive blood cultures can also be seen in Mycobacterium avium, which is usually present in patients with advanced HIV infection [6]. We were initially misled towards the wrong diagnosis as Mycobacterium avium complex on the preliminary test was deemed a contaminant. In instances where obtaining a sputum culture is difficult (such as with our patient), a stool culture offers an alternative method for diagnosing TB, which can be of particular utility when the patient with suspected TB presents with lower gastrointestinal symptoms [16]. Other smear and culture examination sources include gastric lavage, pleural, peritoneal, or pericardial fluid, cerebrospinal fluid, urine, pus from a cold abscess, or bronchoscopic secretions [6]. Tissue biopsy of the affected site(s) can be histopathologically examined, where specimens in the setting of TB demonstrate granulomatous inflammation [15]. The presence of caseating granulomas (granulomas with central "cheese-like" necrosis) is characteristic, but its absence does not exclude miliary TB. This was the case in our patient, who presented with noncaseating granulomas on bone marrow and abdominal fat pad biopsy. Ultimately the gold standard for diagnosing TB is either culture isolating TB or detecting nucleic acids specific to TB (e.g., nucleic acid amplification tests) [17].

Miliary TB can be a challenge to diagnose. The difficulty in identifying the disease can delay prompt treatment, which plays a role in the high rates of morbidity and mortality from this disease. If untreated, miliary TB is fatal within 12 months [6]. Factors such as changes in mental status, meningitis, female sex, dyspnea, advanced age, and a failure to initiate treatment quickly are all associated with a worse prognosis [4,7]. As a consequence, it is imperative to begin treatment early. The availability of antibiotics has markedly improved clinical outcomes. The general approach to treating miliary TB mirrors the traditional six-month regimen of pulmonary TB, consisting of isoniazid, rifampin, and pyrazinamide for two months, followed by isoniazid and rifampin for four months. Ethambutol can be added if resistance to rifampin is suspected, and its continuation is recommended until sensitivity to rifampin is demonstrated [18]. Unfortunately, our patient's nonspecific and uncharacteristic presentation made diagnosing and promptly treating his TB difficult, ultimately resulting in the patient's demise.

## Conclusions

TB, despite diagnostic advances, remains a difficult diagnostic task, as it can mimic multiple other disease processes, have a varied clinical presentation, give rise to a variety of different abnormalities in laboratory tests, and unique radiographic abnormalities, which can be difficult to interpret especially in the background of other illnesses. The challenge is an even further uphill climb in resource-rich settings, where tuberculosis cases are rare. However, this case again emphasizes the dire need for all physicians to be aware of the pathophysiology, clinical presentation, diagnostic approach, and treatment of TB, as it is still a lingering problem in developed countries, especially in the immunocompromised population.
